# Supplementary material for: Predicting visual function by interpreting a neuronal wiring diagram
Source: Nature. 2024 Oct 2;634(8032):113–23. doi: 10.1038/s41586-024-07953-5 (PMC11446822; doi:10.1038/s41586-024-07953-5)
Supplement: Supplementary file 6 — CRF and ERF predictions for individual TmY4 and TmY9 cells. Analogous to Supplementary Data 3, but for TmY target types. Shown are the top four monosynaptic pathways, the strongest pathway passing through each of the top ten intermediary types (ranking from Extended Data Fig. 7), and the trisynaptic pathway Tm1–TmY–Dm3–TmY (see the section entitled Prediction of spatial normalization). [file 41586_2024_7953_MOESM6_ESM.zip › DataS4/TmY9qΓèÑ/720575940622842333.pdf]

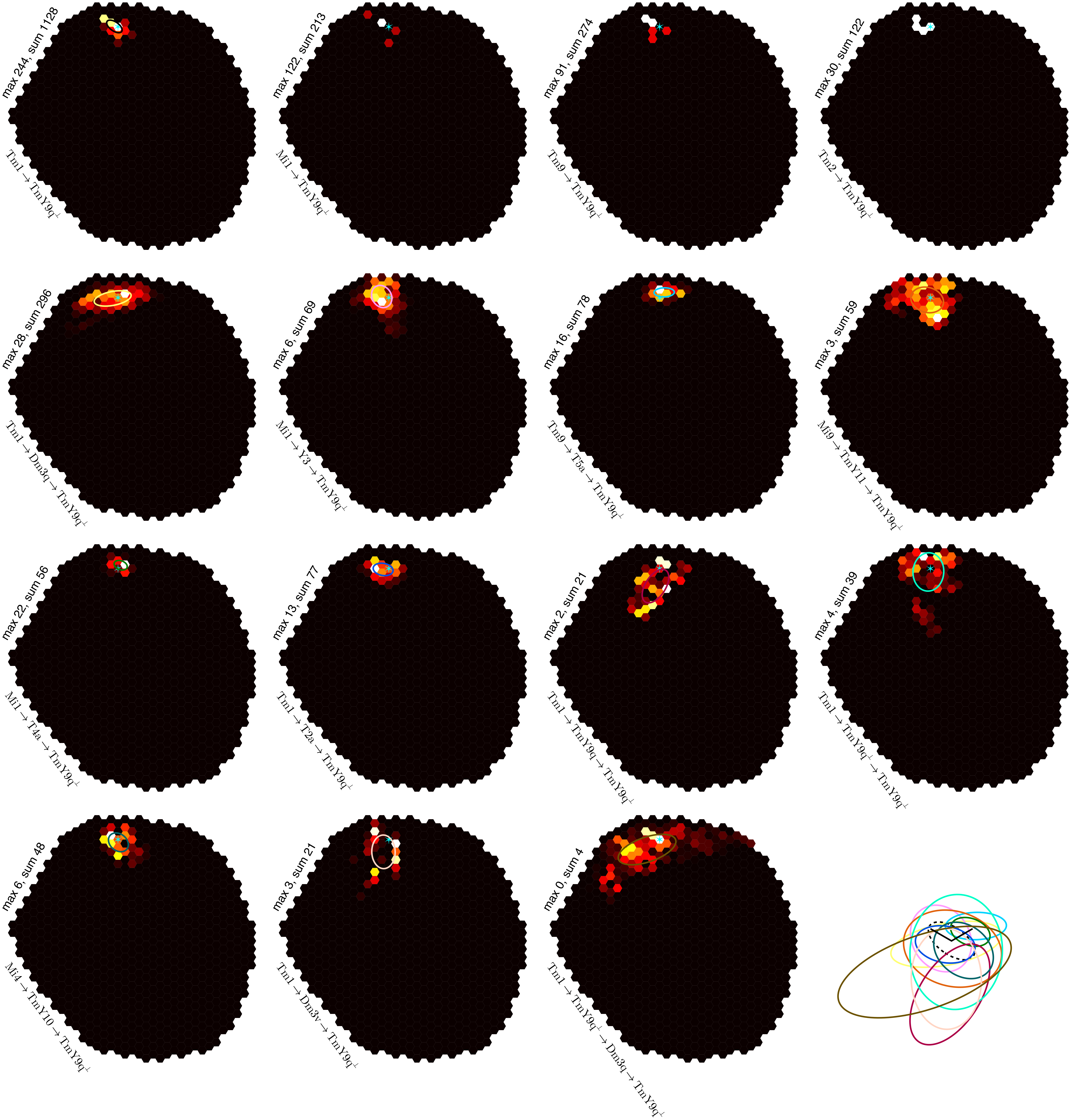
max 244, sum 1128
Tm1 → TmY9q⊥
max 122, sum 213
Mi1 → TmY9q⊥
max 91, sum 274
Tm9 → TmY9q⊥
max 30, sum 122
Tm2 → TmY9q⊥
max 28, sum 296
Tm1 → Dm3q → TmY9q⊥
max 6, sum 69
Mi1 → Y3 → TmY9q⊥
max 16, sum 78
Tm9 → T5a → TmY9q⊥
max 3, sum 59
Mi9 → TmY11 → TmY9q⊥
max 22, sum 56
Mi1 → T4a → TmY9q⊥
max 13, sum 77
Tm1 → T2a → TmY9q⊥
max 2, sum 21
Tm1 → TmY9q → TmY9q⊥
max 4, sum 39
Tm1 → TmY9q⊥ → TmY9q⊥
max 6, sum 48
Mi4 → TmY10 → TmY9q⊥
max 3, sum 21
Tm1 → Dm3v → TmY9q⊥
max 0, sum 4
Tm1 → TmY9q⊥ → Dm3q → TmY9q⊥
